# Substrate Rigidity Controls Activation and Durotaxis in Pancreatic Stellate Cells

**DOI:** 10.1038/s41598-017-02689-x

**Published:** 2017-05-31

**Authors:** Dariusz Lachowski, Ernesto Cortes, Daniel Pink, Antonios Chronopoulos, Saadia A. Karim, Jennifer P. Morton, Armando E. del Río Hernández

**Affiliations:** 1 Cellular and Molecular Biomechanics Laboratory, Department of Bioengineering, Imperial College London, London, SW7 2AZ United Kingdom; 2 Pancreatic Cancer Research Team, CRUK Beatson Institute, Glasgow, G61 1BD United Kingdom

## Abstract

Pancreatic Ductal Adenocarcinoma (PDAC) is an aggressive malignancy characterised by the presence of extensive desmoplasia, thought to be responsible for the poor response of patients to systemic therapies. Pancreatic stellate cells (PSCs) are key mediators in the production of this fibrotic stroma, upon activation transitioning to a myofibroblast-like, high matrix secreting phenotype. Given their importance in disease progression, characterisation of PSC activation has been extensive, however one aspect that has been overlooked is the mechano-sensing properties of the cell. Here, through the use of a physiomimetic system that recapitulates the mechanical microenvironment found within healthy and fibrotic pancreas, we demonstrate that matrix stiffness regulates activation and mechanotaxis in PSCs. We show the ability of PSCs to undergo phenotypic transition solely as a result of changes in extracellular matrix stiffness, whilst observing the ability of PSCs to durotactically respond to stiffness variations within their local environment. Our findings implicate the mechanical microenvironment as a potent contributor to PDAC progression and survival via induction of PSC activation and fibrosis, suggesting that direct mechanical reprogramming of PSCs may be a viable alternative in the treatment of this lethal disease.

## Introduction

Pancreatic Ductal Adenocarcinoma (PDAC) is a highly aggressive malignancy characterised by rapid progression, invasiveness and resistance to treatment^1^. The cancer is almost uniformly lethal with a dismal 5-year survival rate of less than 5%^2^ and a median survival time of 6 months from diagnosis^3^. Despite efforts over the past few decades, conventional treatment approaches such as chemotherapy, radiotherapy, and resection have had little impact on disease progression^4^, owing to the extreme resistance of pancreatic malignancies to all extant treatments^1^. One of the unique and defining features of PDAC is the presence of remarkable stiffness and extensive desmoplasia surrounding the tumour^5^, which is thought to generate a unique microenvironment that facilitates cancer growth^6^, survival^6–9^ and metastasis^10–12^.

Through various *in vivo* and *in vitro* studies^5, 10, 13–17^ pancreatic stellate cells (PSCs) have been identified as the cell type responsible for the production and maintenance of this growth permissive microenvironment. Under normal conditions, these myofibroblast-like cells play a role in maintaining the normal tissue architecture of the pancreas^14^. Upon pancreatic injury, PSCs transition from a quiescent, vitamin A lipid storing phenotype^18^, to an activated state characterized by changes in migratory capacity and an increase in mitotic index and extracellular matrix secretion (ECM)^19^. In health, this ECM remodeling results in wound healing and the subsequent removal of activated PSCs through apoptosis^20^. In pancreatic cancer however, PSC activation is induced and maintained through the release of soluble growth factors and cytokines by cancer cells^14, 21^, resulting in the characteristic stromal ‘reaction’ around the tumour. Once produced, this leads to a vicious cycle of accelerated cancer proliferation and subsequent mitogen production, perpetuating PSC activity^6^.

Given the role this desmoplastic stroma, and particularly PSCs, play in cancer progression and survival, research has accordingly switched to targeting aspects of the tumour microenvironment, such as PSCs and the pronounced fibrosis. Stromal ablation techniques however, have thus far been met with limited and somewhat contradictory results^22, 23^. Unlike stromal depletion strategies, stromal reprogramming is an emerging concept gaining acceptance as an attractive alternative PDAC therapy^24^. Such an approach is supported through a recent report showing that vitamin D analogues are capable of transcriptionally reprogramming pancreatic stellate cells and overall tumour-associated stroma into a more quiescent state, which resulted in reduced tumour volume and an increase in intratumoral gemcitabine^24^.

It is well known that soluble profibrotic factors released from cancer cells activate both local^14, 21^ and distant^25^ PSCs, which migrate from remote sites in the pancreas towards the tumour core. Here, crosstalk between activated PSCs and cancer cells promote PDAC carcinogenesis^6^ and chemoresistance^6–9^. Activated PSCs have also been shown to play a key role in cancer metastasis^11, 12^, participating in the formation of distant metastatic sites through co-migration with cancer cells^11^ and through the creation of ‘tracks’ within tissues, aiding in cancer cell migration^26^. Therefore, there is an urgent, currently unmet need in the field of pancreatic cancer to find therapies that induce PSC deactivation.

Interestingly, efforts thus far seem to overlook any potential role for the mechanical PDAC microenvironment in regulating PSC activity. The fact that PDAC is one of the most fibrotic and stroma-rich malignancies intuitively leads to the idea that extracellular matrix mechanics may play a key role in the development of fibrosis and PDAC progression. Studies that address the influence of mechanical force on PSC-PDAC interactions however, are severely lacking with some exceptions such as a recent study by Weaver and colleagues that has revealed that changes in matrix rigidity associated with PDAC fibrosis has a pronounced effect on the malignant epithelium, accelerating PDAC progression via changes in integrin-mediated mechanosignalling. This leads to the notion that ECM rigidity may also alter the ECM tensional homeostasis to influence the activity of PSCs in the stromal compartment of the tumour, therefore accelerating the development of fibrosis within a positive feedback loop^27^.

In a first attempt to mechanically reprogram PDAC-associated stroma, our group reported that ATRA, an active metabolite of vitamin A, restores mechanical quiescence in PSCs, in an actomyosin dependent manner and inhibiting local cancer cell invasion in 3D organotypic models^28^. Such studies, however, involve analysis of cells cultured on glass – a substrate with rigidity in the order of GPa^29^ – and as such fail to recapitulate a biologically relevant environment. It is a well-known phenomenon that transdifferentiation of PSCs to an active phenotype occurs during culture on glass^30^, however the question of whether or not PSCs possess the ability to mechanically sense the rigidity of their local fibrotic environment and undergo phenotypic transition solely as a result of mechanical stress has never been addressed. Furthermore, whilst the ability of PSCs to chemotactically migrate towards pancreatic neoplasms has been well defined^25^, whether or not PSCs display durotactic behaviours within this microenvironment has not been explored.

Here, through the use of a physiomimetic system that recapitulates the mechanical microenvironment found within healthy and fibrotic pancreas, we show that matrices mirroring rigidities found within fibrotic pancreas activate PSCs, whilst matrices resembling healthy pancreas induce and maintain quiescence in previously activated PSCs. Moreover, activated PSCs were also observed to undergo durotactic migration towards stiffer, fibrotic-like regions; a response previously characterized in fibroblasts^31^, but not reported before in PSCs.

## Results

### Matrigel induces PSC quiescence

**Figure 1:**
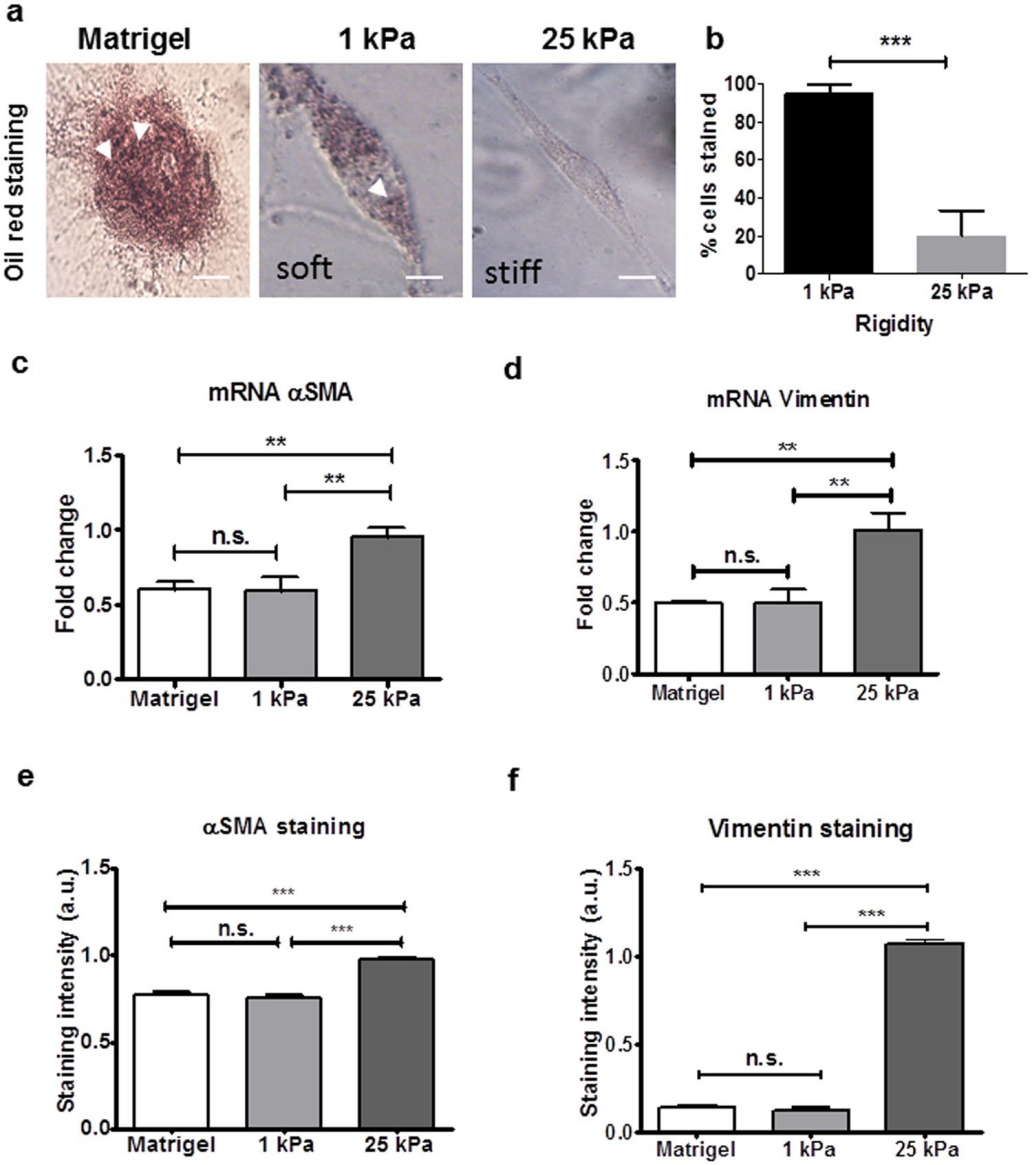
Stiff matrices induce PSC activation. (**a**) Bright-field images of Oil Red O stained PSCs on Matrigel, soft and stiff matrices for 6 days. Scale bar 25 μm. (**b**) Quantification of Oil Red O staining after 24-hour culture on soft or stiff PAA matrices showed a significant reduction in staining levels on stiff matrix when compared to soft matrix, indicating cellular activation almost entirely on stiff matrix rigidities. (**c**,**d**) pPCR mRNA levels of αSMA and vimentin for conditions represented in (**a**). (**e**,**f**) Quantification of staining intensity for αSMA and vimentin for conditions represented in (**a**), images in Supplementary Fig. 3. In all cases, histogram bars represent mean ± SEM. Representative of 3 independent experiments with more than 20 cells analysed in (**b**,**e**,**f**), ***p < 0.001, **p < 0.01.

Transition of quiescent PSCs to an activated myofibroblast-like state is a well-documented phenomenon that occurs upon cell culture^30^. Indeed, all fibroblasts grown in standard culture conditions are myofibroblast by definition, given that contact with the stiff surface of culture flasks triggers the formation of contractile stress fibres^32^. As a result, the assessment of any potential mechano-sensory regulation of PSC activity is not possible using this setup. To address this issue, we sought to implement an *in vitro* model that allows us to culture PSCs in a quiescent state. Transdifferentiated culture-activated PSCs were grown on a layer of Matrigel for 6 days to induce cell quiescence, following an *in vitro* method identified by *Jesnowski et al*.^30^. Matrigel culture resulted in reversion of activated PSCs to a quiescent-like state. Cells lost their spindle morphology and Oil Red staining was used to confirm the presence of cytoplasmic lipid droplets characteristic of PSC quiescence (Fig. 1a).

Our observations are in agreement with previous results described by *Jesnowski et al*.^30^, and confirm that Matrigel culture of activated PSCs results in the reversion of cells to a resting-like state. In addition to resumption of lipid storing ability, cells on Matrigel began to form cell clusters connected by a filamentous network (Supplementary Fig. S1), further mirroring earlier observations by *Jesnowski et al*.^30^. Taken together, these results indicate the ability of Matrigel to revert culture-activated PSCs to a state of quiescence, whilst indicating the matrix surrounding PSCs plays a pivotal role in the maintenance of PSC activation^30^.

### Production of a physiomimetic model recapitulating soft and stiff substrates

The ability of cells to sense and respond to environmental mechanical force is a key determinant in tissue homeostasis^33^. Whilst activation of PSCs in physiological conditions is a well-regulated defined process, the unabated activation leads to sustained fibrosis^34^. Although prior observations are suggestive, there has of yet been no direct demonstration that PSCs are able to adapt behaviour based on the mechanical properties of their substrate. To explore if the exogenous mechanical environment is enough in itself to regulate PSC activity, a physiomimetic model representing soft and stiff tissues was produced.

Polyacrylamide (PAA) gels of varying rigidity – 1 kPa (soft matrix) or 25 kPa (stiff matrix) – were prepared according to Engler’s protocol^35^, through alteration of gel acrylamide/bis-acrylamide ratios (Supplementary Table S1). Cell culture on these synthetic hydrogels requires the coupling of a cell-adhesive matrix protein in order to provide proper cell attachment to the gel surface^35^. Through the use of the substrate-protein crosslinker sulpho-SANPAH^36^, gels were crosslinked with the ECM protein fibronectin (Supplementary Fig. S2), yielding a mechanically tunable, chemically identical PAA hydrogel system, capable of providing a platform upon which to investigate how substrate stiffness regulates PSC behaviour.

### Stiff matrices induce PSC activation

PDAC is intrinsically one of the most fibrotic and rigid human malignancies, ascribed in part, to the dense collagenous stroma that surrounds the neoplasm^10^. To identify if this stiff mechanical microenvironment is enough alone to induce PSC activation, Matrigel-induced quiescent PSCs were seeded onto PAA hydrogels resembling soft (1 kPa) and stiff (25 kPa) tissues, referred hereafter as soft and stiff, respectively. After 24 hours of culture, we used Oil Red staining to identify the presence of any cytoplasmic lipid droplets characteristic of PSC quiescence (Fig. 1a). We observed that quiescent PSCs seeded onto soft matrices retained the ability to store lipid droplets, suggesting maintenance of a resting-like state. Quantification of seeded cell populations revealed a statistically significant difference (p < 0.001) in total Oil Red staining levels between PSCs on soft (95% stained) and those on stiff (20%) hydrogels (Fig. 1b). To further validate our observations, we tested the expression of alpha smooth muscle actin (αSMA) and vimentin, two widely used markers for quiescence in PSCs, at the gene and protein levels^10, 37^. We observed no significance difference in the mRNA levels of αSMA and vimentin of PSCs seeded on Matrigel (standard technique to induce PSCs quiescence) and soft matrices. Conversely, we found a two-fold increase in the mRNA levels of αSMA and vimentin of PSCs seeded on stiff matrices compared to soft matrices and Matrigel (Fig. 1c,d). At the protein level, we observed a similar trend, no significant differences in the expression of αSMA and vimentin between PSCs on matrigel and soft matrix, and a significant increase in both proteins expressions when PSCs were on stiff matrices (Figs 1e,f and S3). We also observed that stiff substrates increase PSC proliferation and fibronectin expression (Supplementary Fig. S4).

Taken together that quiescent PSCs seeded onto stiffer matrices were observed to lose lipid-storing capacity, express the canonical markers characteristic of PSC activation, and increase proliferation & ECM protein production, these observations indicate that substrate stiffness can, per se, induce phenotypic transition of PSCs to a matrix-secreting active state. Serum conditions were kept the same throughout the experiments, indicating that the observed changes occurred irrespective of the presence of any soluble factors.

### Soft matrices induce and maintain PSC quiescence

**Figure 2:**
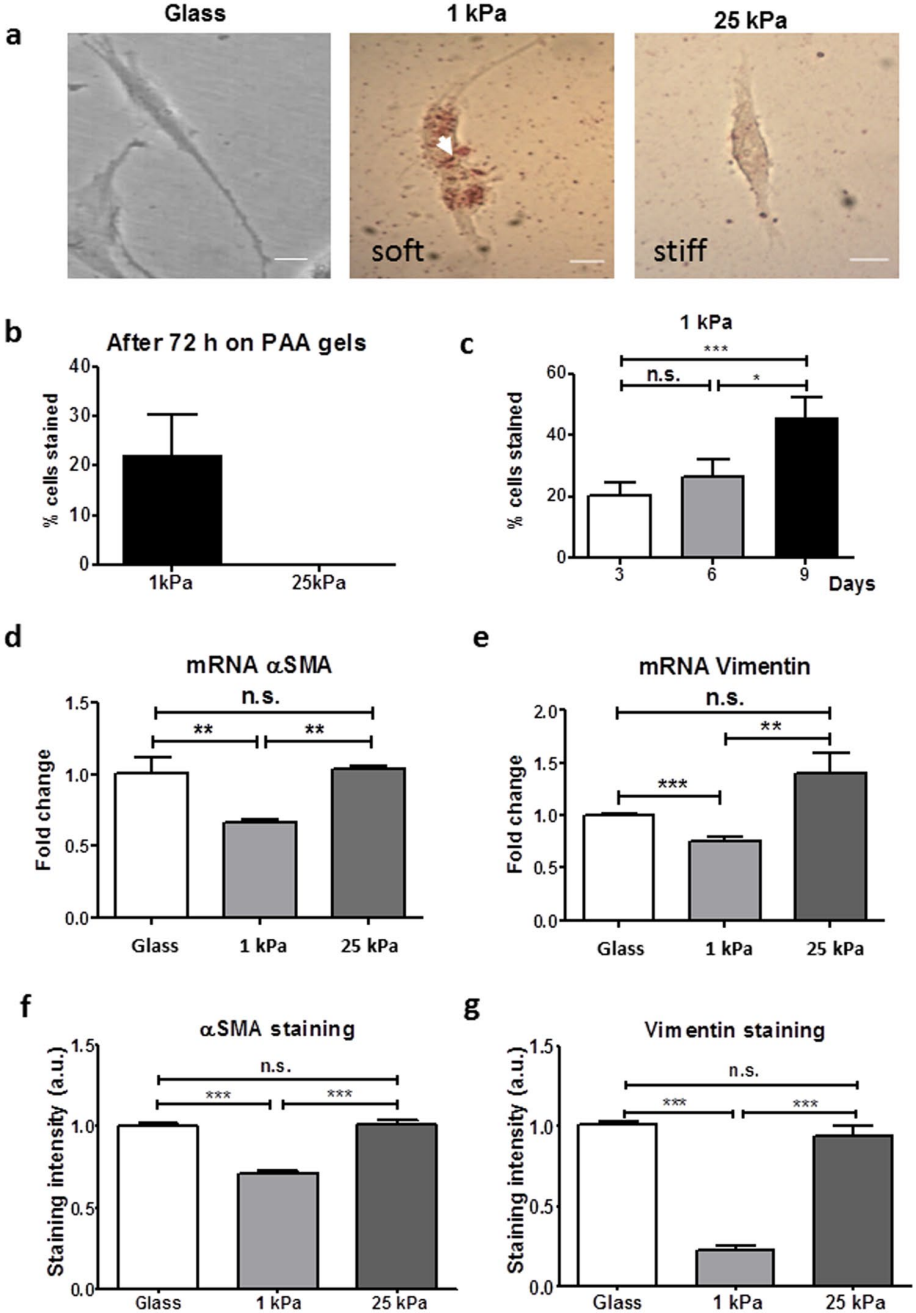
Soft matrices induce PSC deactivation. (**a**) Bright-field images of Oil Red O stained PSCs on glass, soft and stiff matrices for 6 days. Scale bar 25 μm. (**b**) Cell population Oil Red staining levels after 3 days on soft or stiff PAA matrices show that active PSCs on soft matrix begin to revert back to quiescence. Representative of 3 independent experiments with 33 cells analysed. (**c**) Cell population Oil Red O staining levels after 3, 6 and 9 days on soft matrix confirm that culture of active PSCs on soft matrices reverts cells back into a resting state in a time-dependent manner. (**d**,**e**) pPCR mRNA levels of aSMA and vimentin for conditions represented in (**a**). (**f**,**g**) Quantification of staining intensity for αSMA and vimentin for conditions represented in (**a**), images in Supplementary Fig. 5. In all cases, histogram bars represent mean ± SEM. Data are representative of 3 independent experiments and 221 cells analysed in (**c**), *p < 0.05, **p < 0.01, ***p < 0.001, n.s. non-significant.

Many conditions featuring pathological tissue fibrosis occur as a result of sustained myofibroblast activity^18^. This persistent activation is a consequence of the establishment of a mechanical feedback loop, which perpetuates myofibroblast matrix secretion through the sensing and promotion of a stiff microenvironment^28^. Restoring ECM mechanics to normalcy or the ability of the cell to perceive the elevated ECM rigidity is sufficient to terminate the feedback loop and abrogate myofibroblast activity, cells typically undergoing de-differentiation to a quiescent state^32^. To investigate whether PSCs exhibit this mechano-induced state ‘fluidity’, previously glass culture-activated PSCs were transferred and grown on soft or stiff PAA hydrogels for 3 days, with Oil Red staining employed to identify cell phenotypic state. Cells cultured on stiff matrices were shown to remain continually active, with PSCs lacking lipid-storing ability (Fig. 2a). Conversely, PSCs grown on soft matrices began to regain cytoplasmic lipid droplets (Fig. 2a), indicative of a resumption of quiescence.

Quantification of these populations (Fig. 2b) revealed, as expected, the complete absence of any Oil Red staining on stiff matrices (0% stained), indicating a population-wide maintenance of PSC activation. On soft hydrogels however, after 3 days of culture 22% of the previously outright culture-active population had reverted to a state of quiescence; nearly a quarter of cells regaining lipid-storing capacity (Fig. 2b). Given these findings, we next tested whether further prolonged growth on soft matrices would increase population phenotypic transition to a quiescent state. Glass culture-activated PSCs were grown on soft matrices for a total of 9 days, with Oil Red staining of samples occurring in 3-day intervals to assess PSC population quiescence (Fig. 2c). Staining levels at 3 days (21% stained) were in agreement with our earlier observations, with 6 days (26%) and 9 days (46%) yielding a significant increase in population quiescence.

To learn more about the effect of matrix rigidity on PSCs activation, we next investigated the expression of αSMA and vimentin at the gene and protein levels, as markers of PSCs activation. Consistent with our previous observation, the αSMA and vimentin mRNA levels in PSCs seeded onto stiff matrices were not statistically different from those plated on glass; while the expressions of these two markers on PSCs seeded onto soft matrices were markedly suppressed with regard to glass and stiff matrix (50% and 40% reduction for αSMA and vimentin, respectively) indicating the induction of quiescence on PSCs seeded on soft matrices (Fig. 2d,e). We observed the same trend at the protein level for both markers (Figs 2f,g and S5). Furthermore, we also observed that soft substrates induce a decrease in PSC proliferation and fibronectin expression (Supplementary Fig. S6).

**Figure 3:**
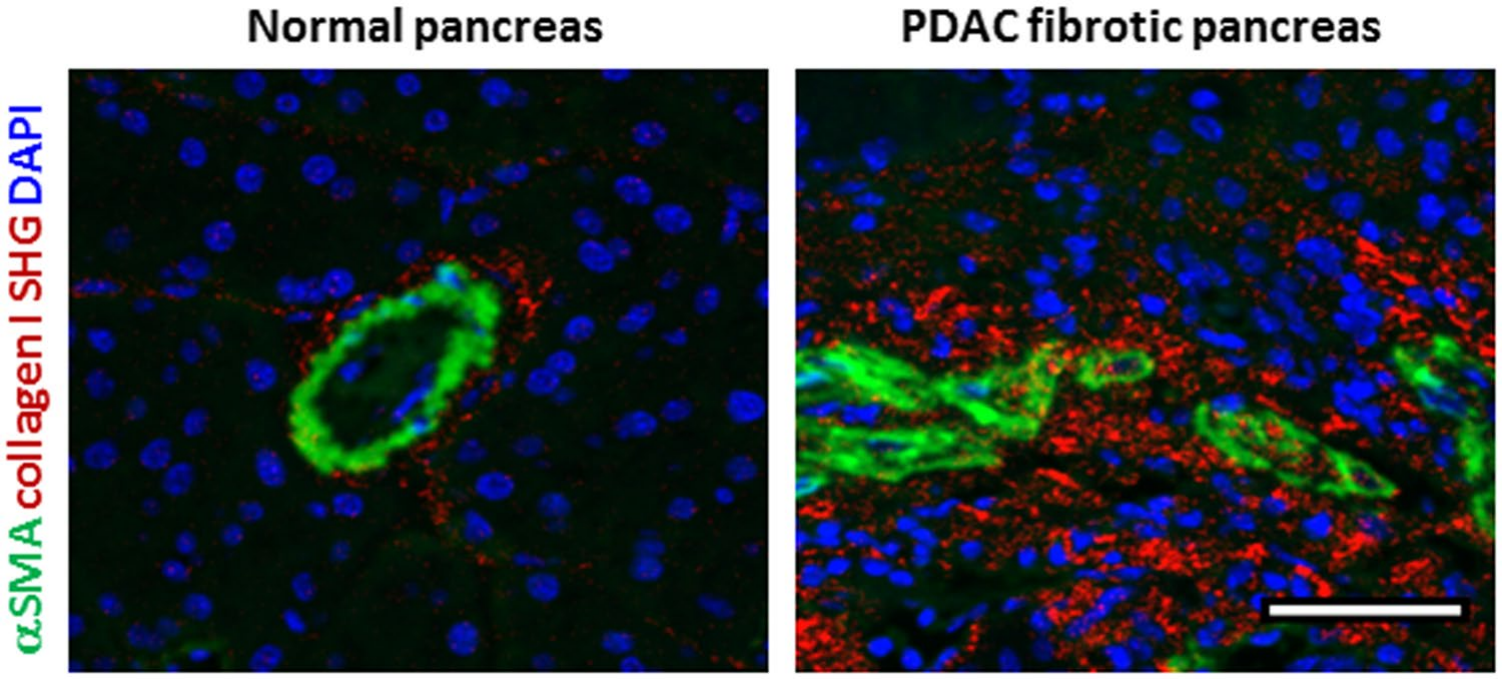
αSMA is highly expressed and co-localises with collagen-I in PDAC tissues but αSMA expression is markedly decreased in normal pancreatic tissues from mice. Immunofluorescence images combined with second harmonic generation (SHG) signal of normal and PDAC tissues from mice. Scale bar 100 μm. Collagen I SHG shown in red.

In order to explore the physiological relevance of our findings, we investigated the activation levels of PSCs in normal (Pdx1-Cre) and fibrotic pancreas associated to PDAC (Pdx-1 Cre, LSL-Kras^G12D/+^, LSL-Trp53^R172H/+^) in mice models. Using immunofluorescence to detect αSMA, and second harmonic generation (SHG) to visualize collagen-I, we observed abundant expression and co-localization of αSMA and collagen-I in PDAC fibrotic tissues. This indicates the presence of active PSCs (αSMA expression used as a surrogate of PSC activation) secreting high levels of ECM proteins. In stark contrast with this, we only observed αSMA expression and collagen deposition in ductal areas of normal pancreas tissues (Fig. 3).

Thus, taken collectively, our data indicate that PSCs are capable of returning to a resting state within a mechanically relevant model of pancreatic fibrosis. Furthermore, these observations directly highlight the importance of the mechanical microenvironment in regulating PSC behaviour, with our results identifying that the stiff microenvironment found within PDAC plays a pivotal role in maintaining the matrix secreting PSC phenotype.

### PSCs exhibit directed migration across a stiffness gradient

**Figure 4:**
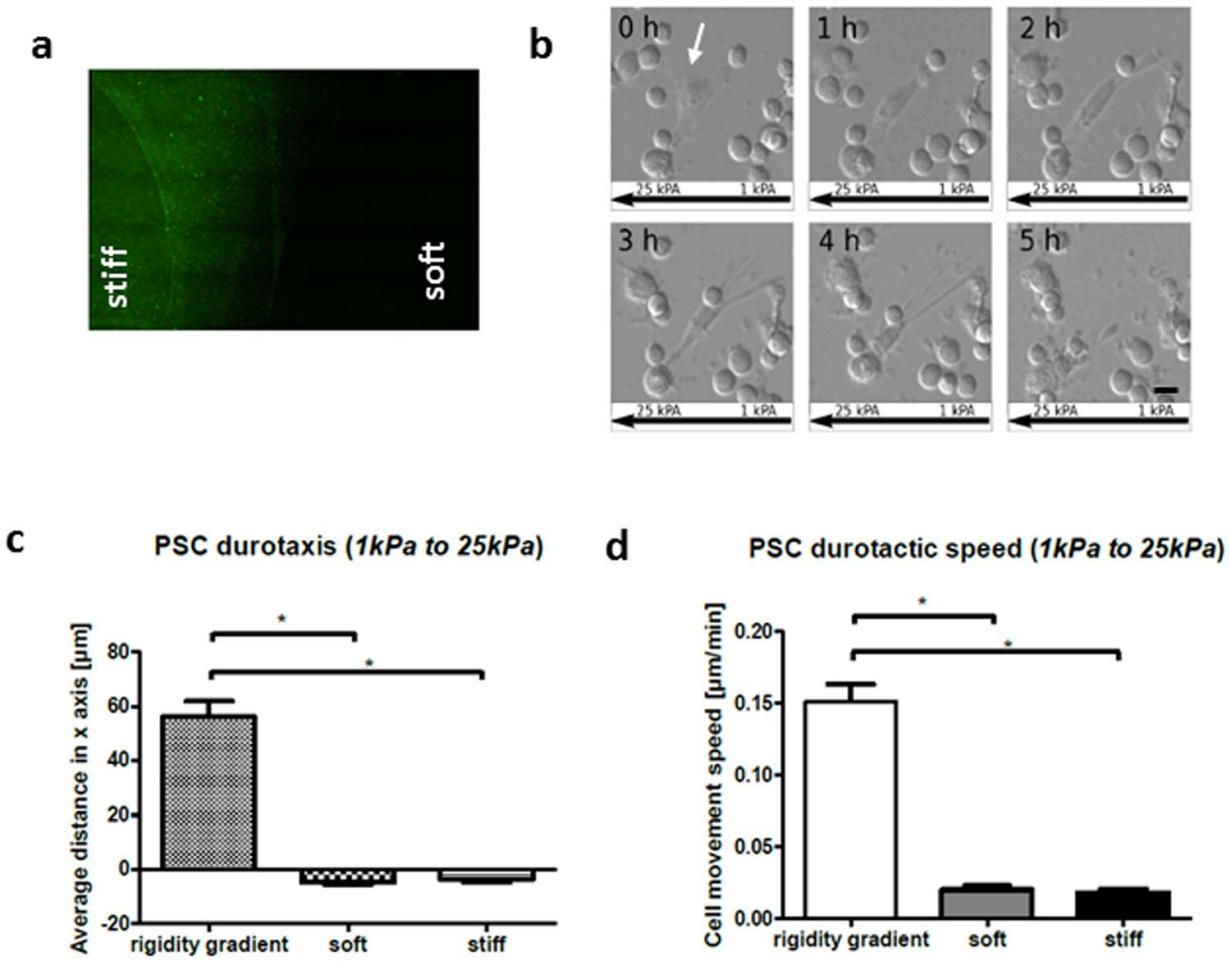
Durotactic response of PSCs. (**a**) Fluorescent image of rigidity boundary between soft (1 kPa) and stiff (25 kPa) PAA matrices. Yellow-green FluoSpheres were embedded into stiff hydrogels. **(b)** Representative example of PSC migration from soft to stiff regions over a 5-hour period. Scale bar 25 μm. Over time the cell (highlighted with a white arrow) moved towards the left (stiffer substrate). **(c)** Average cell migration distance observed at the rigidity boundary, soft region and stiff region of the hydrogel over a 12-hour period. Positive values indicate movement towards higher rigidity, values close to 0 indicate random, undirected movement. PSCs exposed to a stiffness gradient expressed a marked predilection towards stiff substrate, with those exposed to only a single rigidity exhibiting undirected, limited movement. Number of cells analysed per region: boundary – 51; soft – 67; stiff – 78. *p < 0.05. **(d)** Average cell movement speed observed at the rigidity boundary, soft region and stiff region of the hydrogel over a 12-hour period. PSCs exposed to a stiffness gradient exhibit markedly increased migratory speed when compared to PSCs exposed to only a single rigidity. Number of cells analysed per region: boundary – 51; soft – 67; stiff – 78. *p < 0.05, In all cases, histogram bars represent mean ± SEM. Data are representative of 3 independent experiments.

Durotaxis, the ability of cells to detect and move along gradients in substrate stiffness^31^, has been well characterized in fibroblasts^38^. Such migration provides a novel mechanism through which gradients of matrix stiffness can facilitate and drive the progression of fibrosis^39^. Given the differences in matrix rigidity between the fibrotic PDAC microenvironment and normal pancreas, we set out to identify if PSCs possess any durotactic behaviour. We produced a double-rigidity PAA hydrogel system through juxtaposition of functionalized soft and stiff matrices, resembling a model originally used to observe durotactic migration in fibroblasts^38^. Regions of different rigidities were outlined through embedding of fluorescent beads within the stiff region of substrate (Fig. 4a). Culture-active PSCs were seeded onto this dual-rigidity hydrogel and after 30 minutes (to allow attachment), observations were made through time-lapse phase contrast microscopy every 15 minutes over a period of 12 hours. Observations took place simultaneously within the soft, stiff and boundary regions of the hydrogel.

Cell movement distance within each region was calculated by subtraction of initial (0 hours) from final (12 hours) cell position coordinates along the ‘x’ axis, with migration only analysed when movement along the ‘y’ axis, perpendicular to the gradient axis, was 0. This allows for exclusion of factors other than rigidity gradient in affecting cell movement. Positive ‘x’ values indicate a preference of PSCs to migrate towards regions of fibrosis, whilst negative ‘x’ values indicate migration in the opposite direction. Values close to 0 highlight random, undirected cell movement. We observed the preferential ability of PSCs to durotactically migrate from soft to stiff matrices (Fig. 4b and Supplementary Video S1), with quantification of average ‘x’ values outlining a marked predilection of PSCs to migrate from soft to stiff within the boundary region of hydrogels (Fig. 4c). Cells observed within single rigidity regions of the gel (solely soft or stiff), as expected, exhibited random movement along the ‘x’ axis, with PSCs present within these regions displaying no directed motility (Fig. 4c).

PSC movement speed was also assessed as a function of migration, determined in relation to cell movement distance over the experiment duration (12 hours). As to be expected, cells undergoing directed migration within the boundary region exhibited significantly increased migratory speed in comparison to cells residing within single rigidity regions (Fig. 4d).

**Figure 5:**
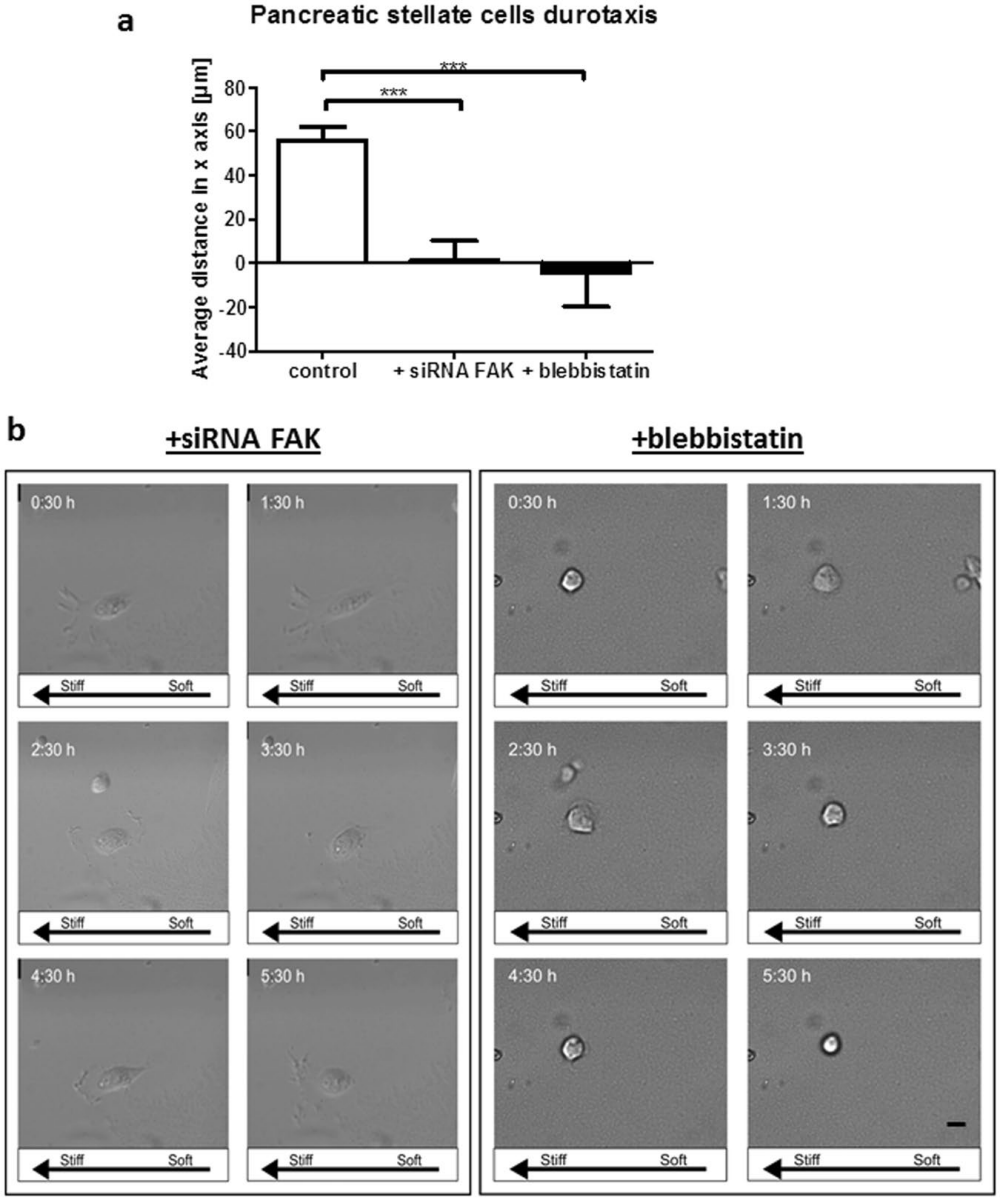
FAK and myosin-II activities are required for durotaxis in PSCs. (**a**) Average cell migration distance at the rigidity boundary over a 12-hour period for control PSCs, PSCs transfected with siRNA for FAK, and PSCs treated with blebbistatin. Positive values indicate movement towards higher rigidity, values close to 0 indicate random, undirected movement. Histogram bars represent mean ± SEM. ***p < 0.001. (**b**) Representative images of PSC migration over a 5 h period. Scale bar 25 μm.

Cell migration and hence durotaxis depend on very tightly coordinated processes of focal adhesion turnover and detachment of the adherent rear edge via myosin-II mediated contractile forces. Interfering with normal spatiotemporal focal adhesion dynamics or cell contractility impairs durotaxis in fibroblasts and mesenchymal stem cells^40, 41^. To learn more about the mechanisms underlying durotaxis in PSCs, we used siRNA against focal adhesion kinase (siRNA FAK), and blebbistatin that inhibits myosin-II ATPase activity and cell contractility. As expected, down regulating FAK or cell contractility profoundly decreased durotaxis in PSCs, evidenced by close to null average of PSCs movement in the x-axis, which is indicative of random non-directed movement (Fig. 5a,b and Supplementary Videos S2 and S3).

Taken together, our data show that PSCs possess the ability to durotactically migrate towards regions of fibrosis within a mechanically relevant model of PDAC. We demonstrate that such motility occurs in the absence of any chemotactic stimuli, highlighting another avenue through which PSCs contribute to the production of desmoplasia around pancreatic neoplasms, whilst providing a potential additional mechanism through which PSCs play a role in cancer metastasis.

## Discussion

PDAC is a highly aggressive malignancy characterised by persistent activation of pancreatic stellate cells (PSCs), resulting in excessive ECM deposition and secretion of soluble factors, which provides both mechanical and biochemical cues that in turn influence all aspects of tumour progression. Furthermore, the tumour-associated fibrosis in PDAC not only impedes intratumoural drug perfusion, but also alters the mechanical microenvironment by increasing matrix stiffness. This can in turn alter force transmission and deregulate the tensional homeostasis of resident PSCs leading to a perpetual cycle of fibrosis and aberrant PSC activation.

Given that activated PSCs are the main effector cells in pancreatic fibrosis, targeting PSCs can offer a novel therapeutic approach to normalise the tumour stroma. In the past, research has primarily focused on identifying soluble profibrogenic and pro-migratory factors – cytokines and growth factors that mediate PSC activation and migration, with most notable examples, transforming growth factor (TGF-β1) and platelet-derived growth factor (PDGF). Matrix stiffness has traditionally been thought of as a manifestation of disease rather than a contributor to fibrosis and as a result little attention was paid so far to the mechanical microenvironment as a stimulus for PSC activation and migration.

It has previously been shown that activated PSCs possess the ability to mechanically activate latent TGF-β stored within the ECM^42^, producing an autocrine feedback loop that independently sustains PSC fibrotic activity^42^. Furthermore, we have previously shown, using a 3-dimensional model of ECM, that activated PSCs apply higher tension on collagen fibres, producing a greater degree of collagen alignment and fibre thickness^43^ that ultimately perpetuates fibrosis and creates the collagen fibre tracks that are used by cancer cells to migrate^26^. Abrogating PSC activation through tuning matrix rigidity, cytoskeletal contractility, or normalising integrin-mediated mechanosensing thus holds the potential to both suppress mechanical activation of latent TGF-β, and change the alignment of ECM architecture that is conducive to cancer cell invasion and survival^44^.

**Figure 6:**
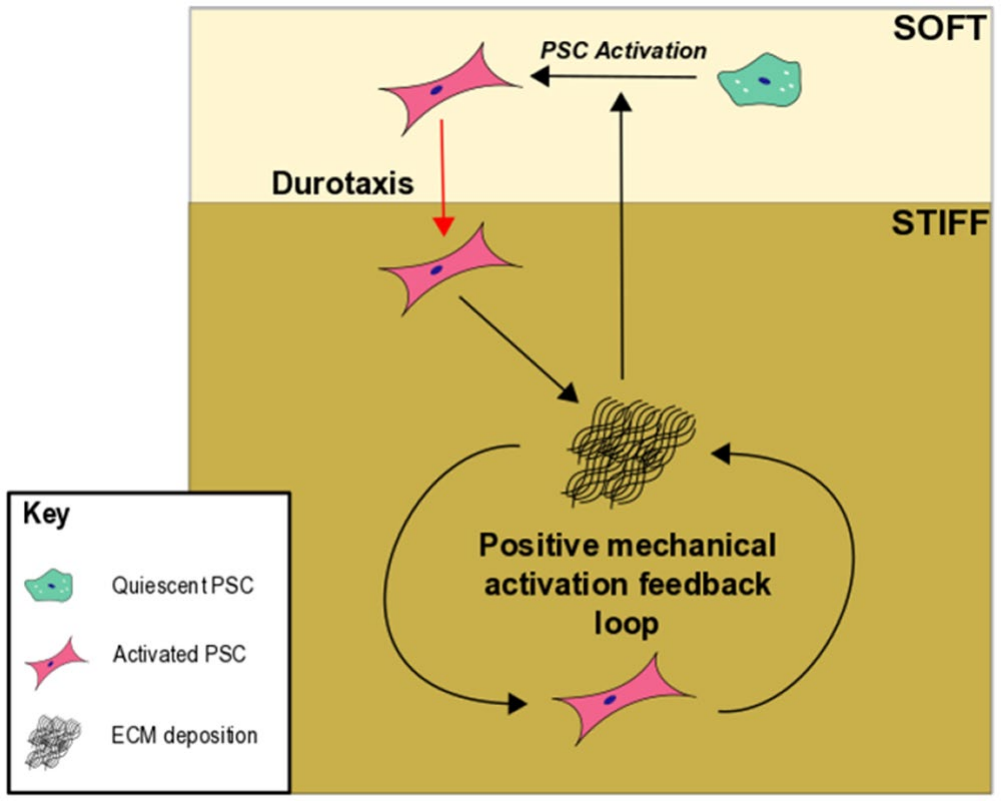
Illustration of PSC mechano-sensory driven regulation within a PDAC microenvironment. Under the effects of activating factors released from nearby cancer cells, local PSCs undergo phenotypic transition to a myofibroblast-like state, characterised by the secretion of vast amounts of ECM, providing a growth permissive environment for the neoplasm. Independently of PSC-cancer cell interactions, the generation of this highly stiff matrix mechanically activates local PSCs through mechanotransduction of the local microenvironment. This leads to increased matrix secretion and further PSC mechano-activation, resulting in the production of a positive mechanical activation feedback loop that produces a continually expanding region of fibrosis around the tumour. Such deposition leads to the generation of a stiffness gradient within the pancreas that is sensed by distant quiescent PSCs, causing them to undergo transition to an active state and begin durotactic migration towards the neoplasm, where upon they contribute to further matrix deposition. This accentuates the ever-growing area of fibrosis around the neoplasm through a vicious cycle of mechanically perturbed PSC activity.

Here, we show a newly identified PSC mechano-sensory regulation within an *in vitro* physiomimetic model of PDAC. Stiff PAA hydrogels, mimicking the PDAC mechanical microenvironment, were shown to induce PSC phenotypic transition to an activated, higher matrix secreting state. This force-mediated activation could explain the perpetuation of established fibrosis. Once resident PSCs are activated through soluble factors released by cancer cells, the matrix secreted by these PSCs creates higher tissue tension in the local microenvironment around the tumour. This increase in stiffness leads to the generation of a positive mechanical feedback loop that both induces and maintains PSC activation (Fig. 6) in the stroma, irrespective of the presence of any soluble factors.

Contrarily, soft matrices, recapitulating healthy pancreas modulus, were demonstrated to induce and maintain PSC quiescence, disproving the idea that apoptosis is solely responsible for termination of PSC activation^45^. The feasibility of stellate cell inactivation is also consistent with previous reports that suggest hepatic stellate cells can revert back to a quiescent state upon resolution of liver fibrosis, although still retaining an intermediate phenotype with enhanced capacity to respond to fibrogenic signals^46^. Such observations may also shed light as to why current therapies targeting the depletion of the myofibroblastic stroma have thus far yielded limited results^22, 23^.

Furthermore, we identified a previously unobserved durotactic response within PSCs, cells preferentially migrating towards regions of fibrosis on a mechanically relevant dual-rigidity PAA hydrogel. Thus, it can be expected that within the PDAC pancreas, such durotactic behaviours complement the already characterised PSC chemotactic movement^25^ in being responsible for the observed increase in activated PSC numbers around the neoplasm^47^. This increase leads to further matrix deposition and subsequent growth of desmoplasia that, in turn, increases durotactic capacity of yet more PSCs, leading to the generation of a positive durotactic feedback loop that complements the aforementioned mechanical loop (Fig. 6).

Taken together, our findings suggest that matrix stiffness can induce myofibroblastic differentiation of PSCs, independently of soluble profibrotic factors (e.g. TGF-β), as well as promote durotactic migration to stiffer fibrotic regions independently of chemotactic stimuli, (e.g. PDGF). Targeting matrix stiffness and mechanotransduction could open new avenues for treatment of pancreatic fibrosis (PDAC and chronic pancreatitis) and fibroproliferative diseases in general. One such avenue includes the recent demonstration that aside from matrix rigidity, cells also sense the length of adhesive ligands that attach them to the matrix^48^. Such information opens up the possibility for engineering applications that make use of longer ‘relaxed’ artificial adhesive tethers that allow PSCs to perceive stiff environments as soft, abrogating fibrotic behaviour.

An alternative and currently more tangible option^28, 42^ is targeted deactivation of PSCs removing the growth permissive microenvironment which surrounds the tumour. Furthermore, through targeting of the mechanosensing properties of PSCs, such treatments have the potential to abrogate PSC mechanical activation of TGF-β^42^, inhibit PSC durotactic migration towards the tumour core and suppress the ability of PSCs to create ‘tracks’ within tissues for further cancer cell invasion^26^. Inhibition of this migratory capacity not only inhibits the crosstalk between PSCs and cancer cells, but also may play an important role in preventing the formation of metastatic niches^28^.

## Materials and Methods

### Cell culture and reagents

Human primary PSCs were purchased from ScienCell Research Laboratories (Carlsbad, USA) and cultured in DMEM/F-12 HAM (Sigma-Aldrich, USA) supplemented with 10% FBS (Gibco, USA), 1% penicillin/streptomycin (Sigma-Aldrich, USA) and 1% Fungizone (Gibco, USA). Cells were tested for contamination and cultured until passage 4–8 was reached.

### Quiescence induction using Matrigel assay

PSC quiescence was induced through culture of cells on Matrigel for 6 days. Corning Matrigel Basement Membrane Matrix, LDEV-free (Scientific Laboratory Supplies, UK) was prepared on ice in a 1:2 ratio with serum-free DMEM/F-12 HAM. Homogenised solution was used to coat sterile positively charged microscope slides/13 mm sterile glass coverslips and left to polymerise for 24 hours at 37 °C. PSCs were then seeded on top of Matrigel and cultured at 37 °C, 5% CO_2_ with media changed every 2–3 days.

### Preparation of polyacrylamide hydrogels of tunable stiffness

Single rigidity PAA hydrogels were prepared through homogenisation of a polymer solution containing: PBS, acrylamide/bisacrylamide (29:1) 40% volume (Sigma-Aldrich, USA), TEMED (Sigma-Aldrich, USA) and 10% APS. Varying hydrogel rigidities were produced through alteration of acrylamide/bisacrylamide amounts (Supplementary Table S1) based on Engler’s protocol^35^. 8 μl (gel attachment to coverslips)/100 μl (microscope slide) drop(s) of desired polymer solution were then transferred to dichlorodimethylsilane (Sigma-Aldrich, USA) treated glass microscope slides before *‘activated’* 13 mm glass coverslips/*‘activated’* glass microscope slide treated with: 0.1 M NaOH, 4% APTES (Sigma-Aldrich, USA) and 2.5% glutaraldehyde (Sigma-Aldrich, USA), were placed on top. Gels were incubated for 45–60 minutes to allow polymerisation before gentle removal from the dichlorodimethylsilane treated microscope slide using a sterile scalpel. Gels were then sterilized under 2 × 30 minutes of UV light and where necessary submerged in PBS and stored at 4 °C.

To produce double rigidity PAA hydrogels suitable for durotaxis analysis, 2.5 μl yellow-green 0.2 μm FluoSpheres carboxylate (Molecular Probes, USA) were added to one of two hydrogel polymer solutions so as to distinguish the boundary between rigidities. FluoSpheres were activated by sonication for 7 seconds. Two 4 μl droplets (one containing FluoSpheres) of varying hydrogel stiffness were placed adjacent to each other on an *‘activated’* glass dish. A dichlorodimethysilane treated coverslip was placed on top and gels allowed to polymerise for 45–60 minutes before gentle removal of coverslip.

To facilitate cell attachment to gels, 50 μl (coverslip)/200 μl (microscope slide) sulfo-SANPAH (SS) (Sigma-Aldrich, USA) solution (0.1 mg SS in 2 μl DMSO/50 μl PBS) was used to covalently bind native human fibronectin (Gibco, USA) to gel surface. Gel surface was covered in SS solution and exposed to 2 × 5 minutes UV light to activate sulfo-SANPAH before excess solution was removed through PBS washing. 50 μl (coverslip)/200 μl (microscope slide) of fibronectin solution (10 μl fibronectin/1 ml PBS) was added to gel surface and gels incubated at RT for 2 hours. Excess fibronectin was then removed with gentle PBS washing. Cells were then added and cultured.

### Oil Red O staining

Oil Red O stock solution was prepared with 60 mg Oil Red O powder (Sigma-Aldrich, USA) dissolved in 20 ml 100% isopropanol and stored at RT in dark. Working solution was prepared by adding 3 parts stock to 2 parts dH_2_0, left to sit for 10 minutes, and then filtered through a 0.2 μm syringe filter. Cells were fixed with 1% PFA, washed with PBS, then incubated with 60% isopropanol for 5 minutes at RT. Isopropanol was removed and cells submerged in Oil Red O working solution for 20 minutes on a dish rocker. Samples were washed with distilled water until clear and stored in distilled water at 4 °C.

### Immunofluorescence

Cells were fixed with 4% PFA, blocked and permeabilised with 2% BSA and 0.1% Triton X-100 (all Sigma-Aldrich, St. Louis, MO, USA) then incubated with primary antibodies (Vimentin M0725 DAKO, Alpha SMA M0851 DAKO, Ki67 ab15580 abcam, Fibronectin ab2413 abcam) 1/100 diluted in 2% BSA/PBS for 1 hour at RT, then washed with PBS and incubated with secondary antibodies (Alexa Fluor® 488 anti-rabbit Life Technologies, USA) and phalloidin (Alexa Fluor® 546, A22283, Life Technologies, USA) 1/500 in PBS for 45 min in dark. Finally the coverslips were mounted with ProLong® Gold Antifade with DAPI (Life Technologies, USA).

### Image acquisition and quantitative analysis

Oil Red O images were taken with a Motic AE31 trinocular inverted microscope by Motic Images Plus 2.0 software using 20x objective. Oil Red O staining was analysed on Matrigel/PAA hydrogels through bright-field microscopy based on the presence/absence of red-stained lipid droplets within cell cytoplasm. Quantification of cell population quiescence per condition was assessed as the number of cells stained positively for Oil Red O within that condition. Immunofluorescent images were taken with Nikon Ti-e inverted microscope by NIS elements software using 40x objective. Immunofluorescent staining was analysed on Matrigel/PAA hydrogels through epifluorescence microscopy based on the mean fluorescence intensity. The immunofluorescent images of pancreas OCT frozen sections and collagen second harmonic generation images were taken with Leica SP5 MP/FLIM upright multiphoton microscope.

### Quantification and analysis of durotaxis on polyacrylamide hydrogels

Durotactic responses of cells were analysed with a Nikon Ti-Eclipse microscope using 20x objective. After cell seeding (control, with 50 µM blebbistatin and with siRNA FAK sc-29310 Santa Cruz Biotechnology transfected with Neon Transfection System, ThermoFisher) onto double rigidity hydrogels, samples were transferred to microscope culture chamber (37 °C, 5% CO_2_) and gently submerged in 5 ml of growth media. Rigidity boundary was identified through yellow-green fluorescence of FluoSpheres. ‘Regions of interest’ (ROI) across the sample were stitched together using NIS elements software to generate a representative image of the hydrogel surface. x- and y-axis were used to define these ROI within the ‘soft’, ‘stiff’, and ‘soft-stiff boundary’ regions of the hydrogel, whilst the z-axis was used to focus the camera onto the surface plane of the gel. A period of 1–2 hours was set to allow cells to fully attach to gel surface before time-lapse phase contrast images were taken every 15 minutes for 12 hours within each designated ROI. Coordinates and distances of cell movement were calculated using the Fiji “Manual Tracking” plugin.

### Real-time quantitative polymerase chain reaction

Total RNA was extracted with RNeasy Mini Kit (Qiagen, 74104) and 1 µg of total RNA was reverse transcribed by High-Capacity RNA-to-cDNA Kit (Applied Biosystems, 4387406) according to manufacturer’s instructions. Q-PCR was performed with SYBR Green PCR Master Mix (Applied Biosystems, 4309155) with 100 ng cDNA input in 20 µl reaction volume. GAPDH expression level was used for normalisation as a housekeeping gene. The sequences were as following: GAPDH: forward-5′ACAGTTGCCATGTAGACC3′, reverse-5′TTTTTGGTTGAGCACAGG3′; a-SMA: forward-5′CATCATGAAGTGTGACATCG3′, reverse-5′GATCTTGATCTTCATGGTGC3′; vimentin: forward-5′GGAAACTAATCTGGATTCACTC3′, reverse-5′CATCTCTAGTTTCAACCGTC3′. All primers were used at 300 nM final concentration. The relative gene expression was analysed by comparative 2^−ΔΔCt^ method.

### Mouse Tissues

Mouse tissues for healthy pancreas (Pdx-1-Cre) and PDAC (Pdx1-Cre; LSL-Kras^G12D/+^ LSL-Trp53^R127H/+^) were obtained from Dr. Jennifer Morton at the Beatson Institute in Glasgow. All experimental protocols were conducted in compliance with the UK Home Office guidelines under license and approved by the local ethical review committee (Beatson Cancer Research UK Institute, Glasgow).

### Multiphoton Microscopy

All SHG images were obtained using a custom built multiphoton microscope incorporating an upright confocal microscope (SP5, Leica) and a mode-locked Ti:Sapphire Laser (Mai Tai, Newport Spectra-Physics). Images of the SHG signal from collagen I were collected using an 820 nm excitation with SHG signal obtained with a 414/46 nm bandpass filter and multiphoton autofluorescence signal obtained with a 525/40 nm bandpass filter. A 25X, 0.95 NA water-immersion objective (Leica) was used to deliver the excitation signal and to collect the SHG emission signal from the sample.

### Statistical analysis

Results were analysed using Prism software. A two-tailed Student’s t-test for unpaired data or ANOVA plus Tukey posthoc test was used to calculate the difference between means, with p-values less than 0.05 considered significant. Single asterisk show *p < 0.05, double asterisk show **p < 0.01, triple asterisk show ***p < 0.001. Data is presented as means, with error bars the standard error of the mean (SEM).

## Electronic supplementary material

Supplementary information

video S1

video S2

video S3
