# Supplementary material for: Substrate Rigidity Controls Activation and Durotaxis in Pancreatic Stellate Cells
Source: Sci Rep. 2017 May 31;7:2506. doi: 10.1038/s41598-017-02689-x (PMC5451433; doi:10.1038/s41598-017-02689-x)
Supplement: Supplementary file 1 — Supplementary information [file 41598_2017_2689_MOESM1_ESM.pdf]

SUPPLEMENTARY INFORMATION

Substrate Rigidity Controls Activation and Durotaxis in Pancreatic Stellate Cells

Dariusz Lachowski[1]*, Ernesto Cortes[1]*, Daniel Pink[1]*, Antonios Chronopoulos[1], Saadia A. Karim[2], Jennifer Morton[2], and Armando E. del Río Hernández[1§]

[1]Cellular and Molecular Biomechanics Laboratory, Department of Bioengineering, Imperial College London, London SW7 2AZ, United Kingdom

[2]Pancreatic Cancer Research Team, CRUK Beatson Institute Glasgow G61 1BD, United Kingdom

*These authors contributed equally

§Corresponding author:

Armando E. del Río Hernández, PhD

Cellular and Molecular Biomechanics Laboratory

Department of Bioengineering

Imperial College London

London, United Kingdom

email: a.del-rio-hernandez@imperial.ac.uk

Supplementary Figure S1

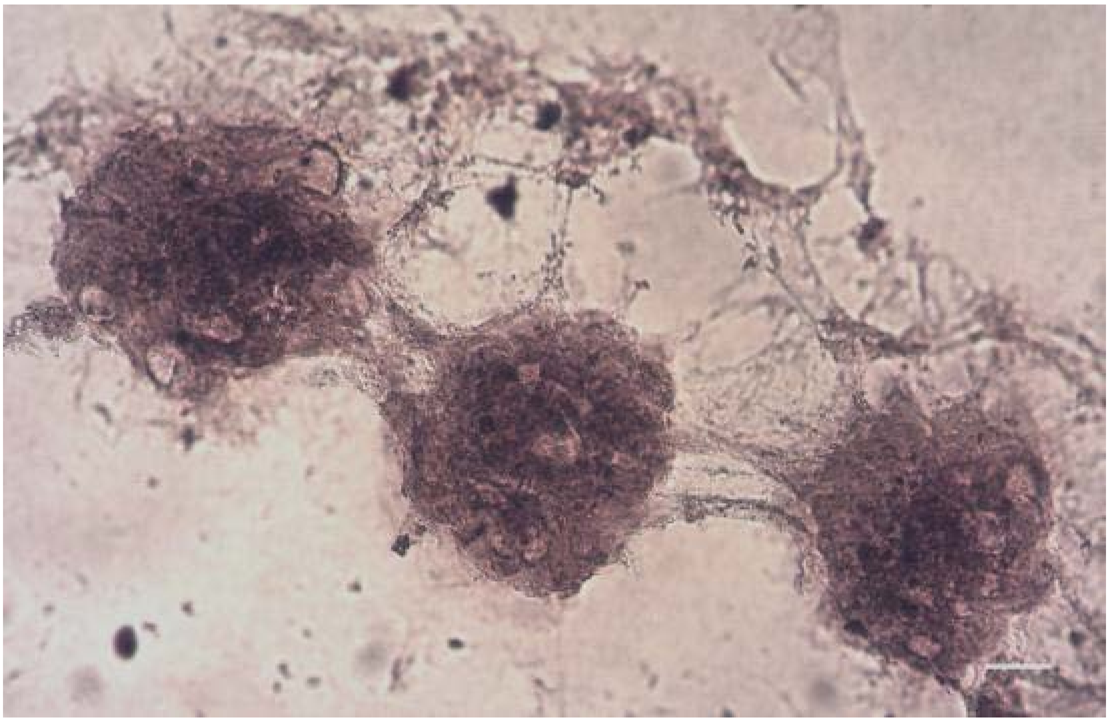

Supplementary Figure 1: Bright field images of Oil Red O stained PSCs cultured on Matrigel for 6 days and showing the clusters connected by a filamentous network characteristic of PSCs quiescence. Scale bar 50 μm

Supplementary Figure S2

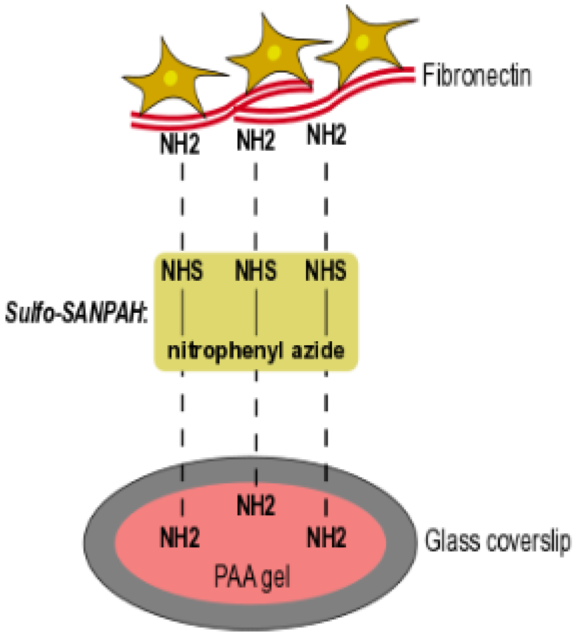

<u>Supplementary Figure 2</u>: Schematic representation of surface preparation for cell attachment on soft and stiff PAA gels**.** Crosslinking of PAA hydrogels with fibronectin through the use of sulfo-SANPAH. sulfo-SANPAH is a heterobifunctional crosslinker containing an amine-reactive NHS and a photoactivatable nitrophenyl azide. Upon UV exposure, this nitrophenyl azide forms a nitrene group that binds to NH_2 groups within the hydrogel, leaving a NHS group free at the gel surface to allow binding of fibronectin.

Supplementary Figure S3

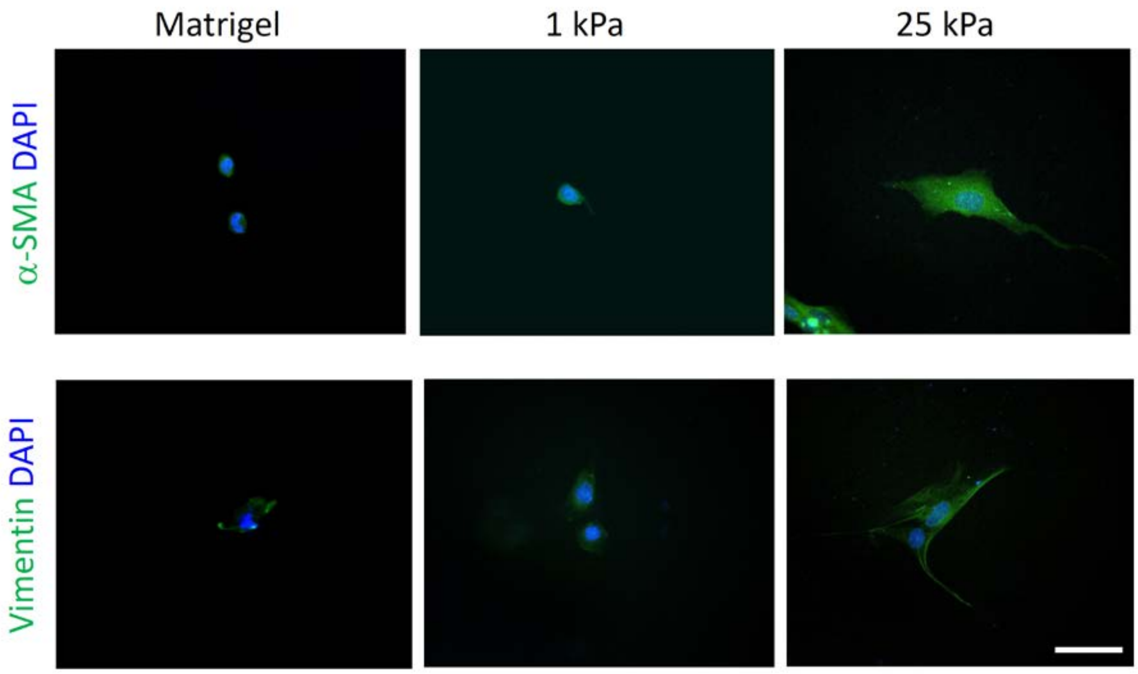

<u>Supplementary Figure 3</u>: Immunofluorescent images of aSMA and vimentin of PSCs seeded on matrices represented in Fig. 1a. Scale bar 50 μm. Quantification in Fig 1e,f.

Supplementary Figure S4

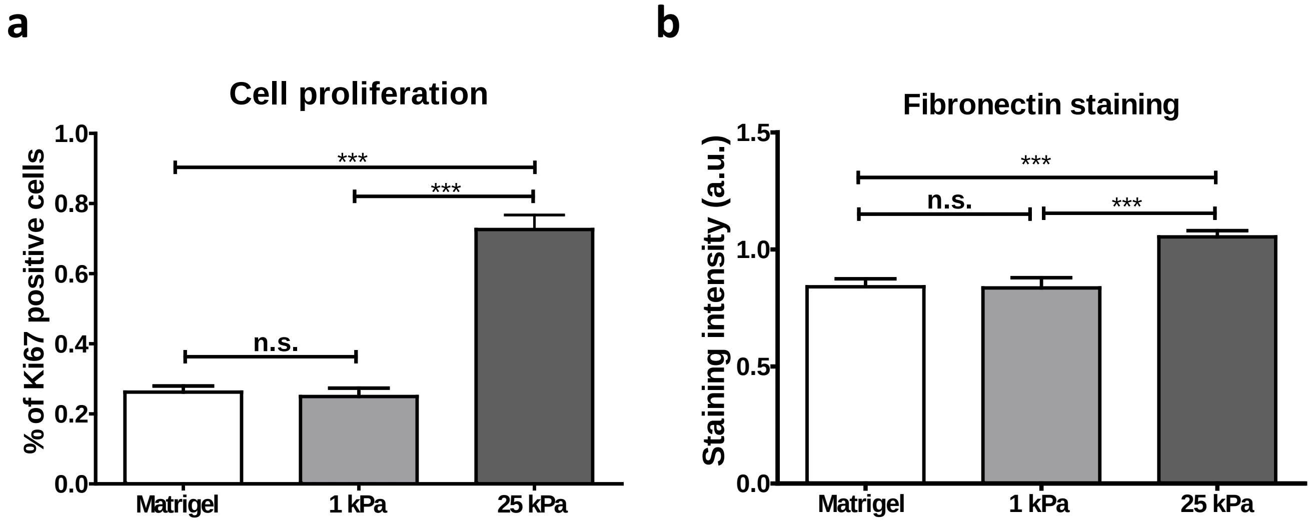

Supplementary Figure 4: **Mechanical activation of PSCs by stiff substrates increases cell proliferation and fibronectin production. (a, b)** Quantification of ki67 positive cells and fibronectin immunofluorescent staining as markers of cell proliferation and ECM production, respectively. Histogram bars represent mean ± SEM. Representative of 3 independent experiments. (Anova and Tukey posthoc test) *** p<0.001.

Supplementary Figure S5

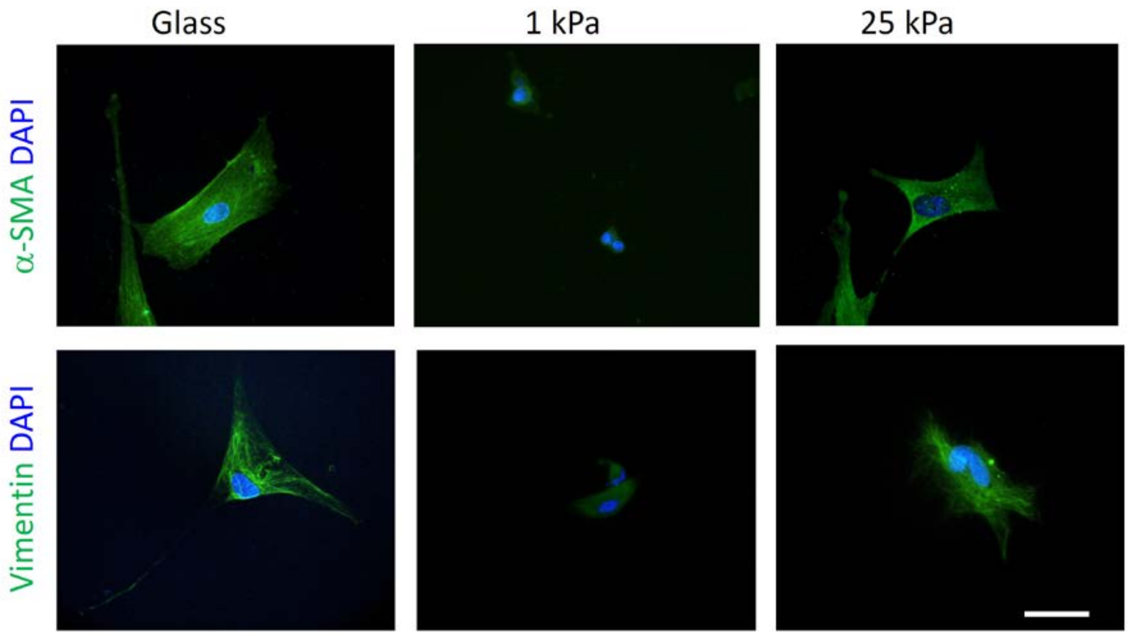

<u>Supplementary Figure 5</u>: Immunofluorescent images of αSMA and vimentin of PSCs seeded on matrices represented in Fig. 2a. Scale bar 50 μm. Quantification in Fig 2 f,g.

Supplementary Figure S6

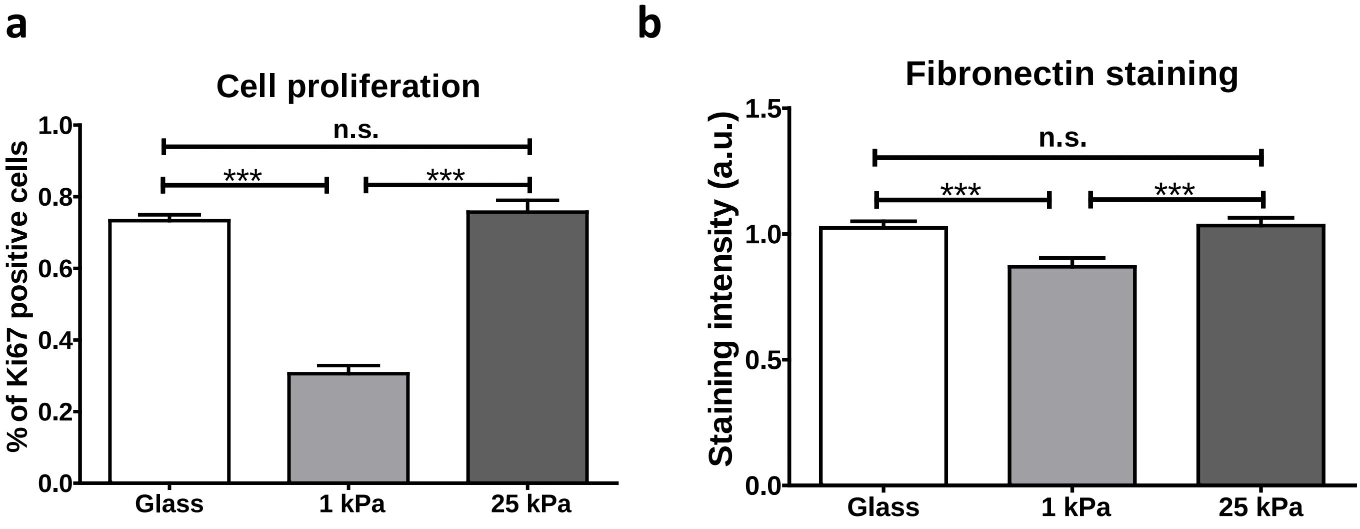

Supplementary Figure 6: Mechanical deactivation of PSCs by soft substrates decreases cell proliferation and fibronectin production. (a, b) Quantification of ki67 positive cells and fibronectin immunofluorescent staining as markers of cell proliferation and ECM production, respectively. Histogram bars represent mean ± SEM. Representative of 3 independent experiments. (Anova and Tukey posthoc test) *** $p<0.001$.

Supplementary Table S1: Reagent proportions required to obtain specific PAA hydrogel rigidities.

Stiffness (kPa)	Total volume (µl)	PBS (µl)	APS (µl)	TEMED (µl)	acrylamide/bisacrylamide (29:1) 40% vol (µl)
1.3	500	461.6	2.5	1	34.9
25.5	500	371.2	2.5	1	125.3

Supplementary Video S1

This video represents the durotactic movement (towards the stiff region) of one pancreatic stellate cell in the boundary region between a soft (right side) to a rigid substrate (left). The total video represents 6 h in real time.

Supplementary Video S2

This video represents the random undirected movement of one pancreatic stellate cell (siRNA FAK) in the boundary region between a soft (right side) to a rigid substrate (left). The total video represents 6 h in real time.

Supplementary Video S3

This video represents the random undirected movement of one pancreatic stellate cell (treated with blebbistatin) in the boundary region between a soft (right side) to a rigid substrate (left). The total video represents 6 h in real time.
